# An Xist-activating antisense RNA required for X-chromosome inactivation

**DOI:** 10.1038/ncomms9564

**Published:** 2015-10-19

**Authors:** Mrinal K. Sarkar, Srimonta Gayen, Surinder Kumar, Emily Maclary, Emily Buttigieg, Michael Hinten, Archana Kumari, Clair Harris, Takashi Sado, Sundeep Kalantry

**Affiliations:** 1 Department of Human Genetics, University of Michigan Medical School, Ann Arbor, Michigan 48109, USA; 2 Department of Advanced Bioscience, Graduate School of Agriculture, Kinki University, 3327-204 Nakamachi, Nara 631-8505, Japan

## Abstract

The transcriptional imbalance due to the difference in the number of X chromosomes between male and female mammals is remedied through X-chromosome inactivation, the epigenetic transcriptional silencing of one of the two X chromosomes in females. The X-linked Xist long non-coding RNA functions as an X inactivation master regulator; Xist is selectively upregulated from the prospective inactive X chromosome and is required in *cis* for X inactivation. Here we discover an Xist antisense long non-coding RNA, XistAR (Xist Activating RNA), which is encoded within exon 1 of the mouse *Xist* gene and is transcribed only from the inactive X chromosome. Selective truncation of XistAR, while sparing the overlapping Xist RNA, leads to a deficiency in Xist RNA expression in *cis* during the initiation of X inactivation. Thus, the *Xist* gene carries within its coding sequence an antisense RNA that drives Xist expression.

## Abstract (web summary)

The X-chromosome linked long non-coding RNA, Xist, is a master regulator of the X inactivation. Here, the authors report that XistAR, an Xist anti-sense long non-coding RNA encoded within the mouse *Xist* gene and transcribed only from the inactive X chromosome, regulates *Xist* expression.

## Graphical abstract

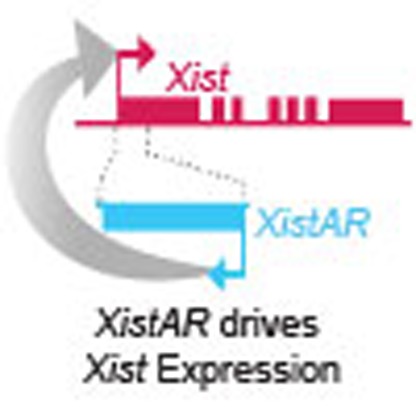

In a shared nucleoplasm, one X chromosome of an identical pair undergoes transcriptional inactivation in mammalian females early during embryonic development. Once inactivated, replicated copies of the inactive X chromosome are transmitted as inactive through many rounds of cell division^1^. For both of these reasons, X inactivation is a model of epigenetic regulation. The Xist long non-coding (lnc) RNA is preferentially upregulated from the prospective inactive X and physically coats that X chromosome at the onset of X inactivation^2^,^3^,^4^. Xist RNA coating is believed to recruit protein complexes that then execute gene silencing on the inactive X (refs 5, 6). How Xist is selectively induced from only one of the two identical X chromosomes is the subject of much debate^7^,^8^,^9^,^10^,^11^,^12^,^13^.

Here we describe the discovery of a novel Xist antisense transcript that is embedded within exon 1 of Xist. This transcript is co-expressed with Xist from the inactive X chromosome. On truncation of the antisense transcript, Xist induction is diminished by ∼90%, resulting in defective X-linked gene silencing on the inactive X. Thus, an Xist antisense RNA activates *Xist* in *cis* and is required for X inactivation.

## Results

### Expression of a novel antisense RNA from the Xist locus

We previously derived F1 hybrid male and female mouse trophoblast stem (TS) and extra-embryonic endoderm (XEN) cell lines deficient in the expression of the Xist antisense transcript Tsix^14^. TS and XEN cells represent progenitors of the extra-embryonic lineages that contribute to the placenta and the yolk sac, respectively. Both cell types undergo imprinted X inactivation resulting in silencing of genes on the paternal X chromosome^15^,^16^,^17^,^18^,^19^. In these cell lines, Xist is normally expressed exclusively from the paternal X chromosome and Tsix from the maternal X chromosome^14^.

**Figure 1:**
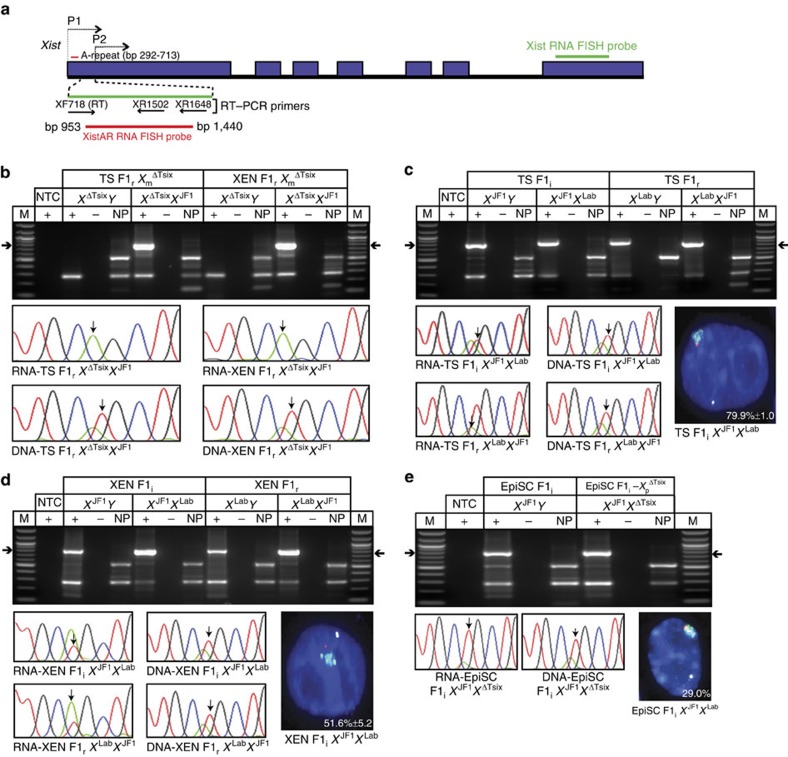
Expression of a novel Xist antisense transcript from the inactive X chromosome. (**a**) Schematic representation of the Xist locus and the location of the primers and RNA FISH probes used. P1 and P2 are start sites of two distinct Xist isoforms^30^,^31^. (**b**) Detection of an Xist antisense transcript from the inactive paternal X chromosome (*X*_p_) in F1 hybrid TS and XEN cell lines lacking expression of the Xist antisense transcript Tsix (*X*^ΔTsix^*X*^JF1^) from the maternal X chromosome (*X*_m_) by strand-specific RT–PCR. Sanger sequencing of cDNAs from females reveals an *X*_p_-specific SNP at bp 804 in Xist exon 1. Genomic DNAs from the same cell lines displays nucleotides from both Xs. (**c**,**d**) Strand-specific RT–PCR amplification of Xist antisense RNAs in wild-type (WT) F1 hybrid (F1_i_, initial cross; F1_r_, reciprocal cross) TS (**c**) and XEN (**d**) cell lines. Sanger sequencing of cDNAs detects SNPs from both Xs in females. The active *X*_m_ expresses Tsix; the inactive *X*_p_ expresses the novel Xist antisense transcript XistAR. (**e**) Strand-specific amplification of XistAR in an *X*^ΔTsix^*X*^JF1^ EpiSC line with biased inactivation of the Tsix-mutant *X*_p_. Sanger sequencing of the cDNAs detects Xist antisense expression from both Xs. As in **c**,**d** while Tsix is expressed from the active *X*_m_, XistAR is expressed from the inactive *X*_p_. M, marker; NTC, no template control; +, reaction with reverse transcriptase (RT); −, reaction without RT; NP, ‘no primer’ control RT reaction without added primer in the RT step but with primers included in the PCR step to exclude reverse transcription of the sense Xist RNA via cell intrinsic primers^20^. Strand-specific RNA FISH detection of XistAR (red), Xist (green) and Atrx (white) RNAs in TS, XEN and EpiSC cell lines. Atrx RNA marks the active X chromosome. Nuclei are stained blue with 4,6-diamidino-2-phenylindole. Three different TS and XEN cell lines and one EpiSC line were stained and >100 nuclei counted in each cell line. Numerical values in the images indicate the percentage of nuclei that display an antisense signal adjacent or overlapping with Xist RNA coat; ±, s.d.

To confirm that the cell lines lacked Tsix expression, we performed strand-specific PCR with reverse transcription (RT–PCR) on RNA purified from Tsix-mutant TS and XEN cell lines. We excluded amplification of genomic DNA in reactions lacking reverse transcriptase in all sets of RT–PCRs (Fig. 1b–e). As an additional negative control, we included reactions lacking primers in the reverse transcription (RT) step. The presence of amplified cDNAs in such reactions suggested that cell intrinsic primers in purified RNAs can function to reverse transcribe RNAs^20^ (Fig. 1b–e). On Sanger sequencing, these cDNAs proved to be nonspecific. To varying degrees, the same amplified cDNAs were also detected in the test RT–PCR samples containing exogenously added RT and PCR primers (Fig. 1b–e). To minimize the spurious RT and PCR amplification by cell intrinsic primers^20^, including, importantly, of the sense Xist RNA, we implemented a modified RT–PCR protocol using tagged RT primers designed to specifically reverse transcribe the antisense transcript (see Methods; primer positions outlined in Fig. 1a). As expected, the test *X*^ΔTsix^*Y* male TS and XEN samples did not display specific amplification of Tsix (Fig. 1b). *X*^ΔTsix^*X* female TS and XEN cells, however, unexpectedly showed a band of the size expected of the Tsix amplicon (Fig. 1b). On Sanger sequencing, the cDNA in fact matched the Tsix sequence.

Expression in mutant female but not male cells suggested that the transcript originated from the inactive paternal X chromosome and not the active maternal X chromosome, as would be expected of Tsix in these cells^14^. We therefore sought to conclusively determine which of the two X chromosomes is the source of the antisense transcript. The two Xs in our F1 hybrid cells are derived from divergent strains of mice and harbour numerous single nucleotide polymorphisms (SNPs) in the *Xist*/*Tsix* sequence. Whereas the X chromosome harbouring the intact Tsix locus is derived from the *Mus molossinus* JF1 strain (*X*^JF1^), the *X*^ΔTsix^ X chromosome is inherited from a *Mus musculus* laboratory strain^14^. We exploited a known SNP in the RT–PCR amplicon to distinguish which X chromosome transcribes the antisense RNA by Sanger sequencing. We found that in both TS and XEN cells, the inactive paternal X chromosome is indeed the source of the transcript (Fig. 1b).

We next assayed Xist antisense expression in wild-type (WT) F1 hybrid male and female TS and XEN cells. To distinguish parent-of-origin-specific from strain-specific bias in expression, we also tested F1 hybrid TS and XEN cells generated from the reciprocal parental cross^14^. In both sets of cells, while one X is the *Mus molossinus*-derived *X*^JF1^ X chromosome, the other X is derived from a *M. musculus* laboratory strain (*X*^Lab^), as in Fig. 1b (ref. 14). TS and XEN cells derived from both the initial (F1_i_) and reciprocal (F1_r_) crosses displayed the specific amplification of antisense RNA(s) in males as well as females (Fig. 1c,d). SNP profiling of the amplified cDNAs demonstrated that Xist antisense transcription occurs from both the maternal (active) and paternal (inactive) X chromosomes in females (Fig. 1c,d). Since Tsix is expressed from the maternal X in the amplicon assayed, the Xist antisense transcript originating from the paternal X is distinct from Tsix, in agreement with Fig. 1b. We termed this novel inactive X-specific Xist antisense transcript XistAR (Xist Activating RNA; see below).

We also independently confirmed XistAR expression from the inactive X by strand-specific RNA fluorescence in situ hybridization (FISH). A single-stranded RNA FISH probe designed to hybridize specifically to Xist antisense transcripts detected a signal that coincided with or was adjacent to the Xist RNA-coated inactive X chromosome in a subset of nuclei in both TS and XEN cell lines (probe location shown in Fig. 1a). The probe unexpectedly did not detect Tsix RNA on the maternal X chromosome in the cells, potentially owing to its lower expression in these cells.

We next tested if the third primary developmental lineage from the early mouse embryo, the epiblast, also expressed XistAR. The pluripotential epiblast cells generate all the cells of the embryo proper and give rise to epiblast stem cells (EpiSCs)^19^,^21^,^22^. Both the epiblast and EpiSCs undergo random inactivation of either the maternal or the paternal X chromosome in individual cells^23^,^24^,^25^. In a randomly inactivated population of cells, however, it is not possible to deduce allele-specific expression of X-linked genes by RT–PCR. We therefore utilized EpiSCs in which X inactivation was biased absolutely in favour of one of the two Xs by assaying XistAR expression in an F1 Tsix-heterozygous EpiSC line^23^. In this hybrid EpiSC line, the *M. musculus*-derived *X*^ΔTsix^ is the inactive X in all cells, while the *M. molossinus*-derived *X*^JF1^ is the active X^23^. We again detected an Xist antisense transcript of the expected size in females but not in males, and only from the inactive *X*^ΔTsix^ in females (Fig. 1e). We confirmed inactive X-specific XistAR expression by RNA FISH in WT EpiSCs (Fig. 1e). Thus, stem cells of all three lineages of the early embryo express XistAR only in females and exclusively from the inactive X.

**Figure 2:**
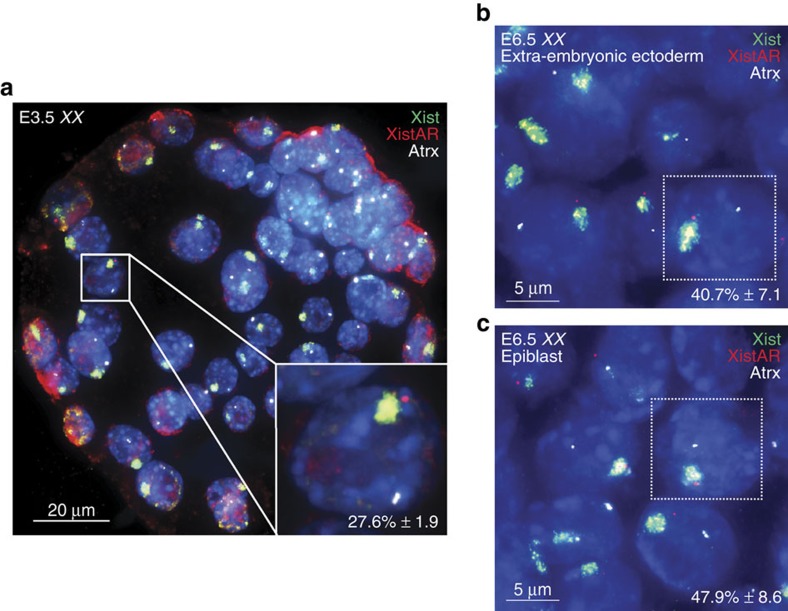
XistAR expression in embryos. Detection of XistAR in female embryonic day (E) 3.5 blastocyst embryos (**a**), E6.5 extra-embyonic ectoderm (**b**) and epiblast cells (**c**) by strand-specific RNA FISH. Numerical values in the images indicate the percentage of nuclei that display an antisense signal adjacent to or overlapping with Xist RNA coat. *n*=4 E3.5 embryos; *n*=3 E6.5 embryo-dissected extra-embryonic ectoderm and epiblast; ±, s.d.

We then wished to know if XistAR RNA is detected from the inactive X in cells of the developing embryo itself. We therefore assayed the expression of XistAR by strand-specific RNA FISH in embryonic day (E) 3.5 mouse blastocysts, which undergo imprinted inactivation of the paternal X in all cells^26^. A substantial percentage of E3.5 nuclei (∼28%) displayed XistAR signal adjacent to or overlapping with Xist RNA coat (Fig. 2a). We similarly tested E6.5 extra-embryonic ectoderm cells, which maintain imprinted X inactivation^17^, and observed XistAR expressed from the inactive X in ∼40% of the nuclei (Fig. 2b). We also found that ∼48% of the randomly inactivated E6.5 epiblast cells displayed inactive X-specific XistAR expression (Fig. 2c). Thus, both in stem cell lines and in the embryo, XistAR is expressed from the inactive X chromosome. That XistAR was not detected in all nuclei may be explained by its low expression or by inefficient probe hybridization.

### Structure of *XistAR*

**Figure 3:**
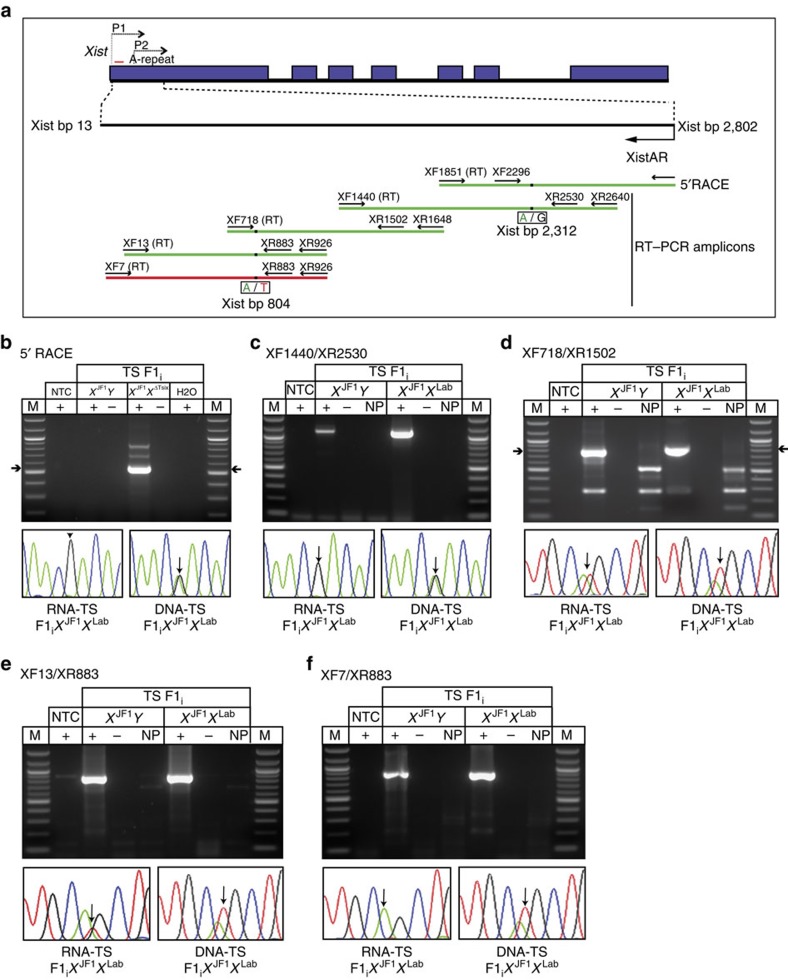
Mapping of *XistAR*. (**a**) Schematic representation of 5′ RACE and RT–PCR strategy to delineate the structure of *XistAR* in WT F1_i_ hybrid TS cells. SNPs that distinguish the maternal and paternal X-chromosome alleles (*X*_m_ and *X*_p_, respectively) are shown under the amplicons. Green amplicons, detection of XistAR expression from the inactive X. Red amplicon, lack of XistAR detection. (**b**) Mapping of 5′ end of XistAR to bp 2,802 of Xist by 5′ RLM-RACE. Sanger sequencing of the major amplified cDNA detects *X*_p_-specific SNPs. Amplified genomic DNA displays SNPs from both alleles. (**c**–**f**) Mapping of *XistAR* by overlapping RT–PCRs. All amplicons except XF7/XR883 detect XistAR. The 3′ end of XistAR therefore maps to ∼Xist bp 13. In **c** Sanger sequencing demonstrates primary amplification of XistAR, but not the Xist antisense transcript Tsix. The PCR primers XR2640 and XR2530 in this amplicon map to intron 3 of Tsix, and thus do not amplify spliced Tsix RNA. The low-level amplification in males is presumptive unspliced Tsix RNA. In **d**,**e** Sanger sequencing detects SNPs from both Xs, since both XistAR and Tsix are reverse transcribed and amplified. In **f**, Sanger sequencing detects amplification of Tsix but not XistAR. M, marker; NTC, no template control; +, reaction with reverse transcriptase (RT); −, reaction without RT; NP, ‘no primer’ control RT reaction without added primers.

**Figure 4:**
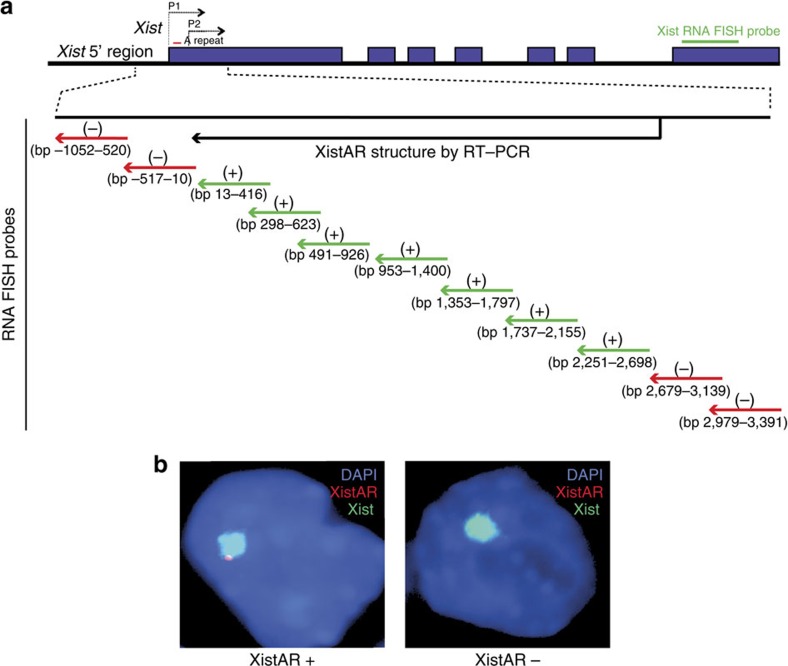
Mapping of *XistAR* by RNA FISH. (**a**) Schematic representation of the *Xist* locus and the position of strand-specific RNA FISH probes used to test XistAR expression in TS cells. Probes depicted by green arrows detect *XistAR* expression, whereas probes represented by red arrows do not. (**b**) Representative nuclei with or without XistAR expression. XistAR RNA (red); Xist RNA (green); blue, 4,6-diamidino-2-phenylindole (DAPI) staining of nuclei.

We next defined the structure of *XistAR* by 5′ RNA ligase mediated-rapid amplification of cDNA ends (RLM-RACE) and by RT–PCR (Fig. 3a). The 5′ RLM-RACE exploits the ^7m^G cap at the 5′ end of RNA polymerase II-transcribed RNAs to precisely map the 5′ end of RNAs^27^,^28^. Using total RNA from TS cells, we thereby mapped the 5′ end of XistAR RNA to base pair (bp) 2,802 of *Xist*, which is in exon 1 (Fig. 3b). We attempted to map the 3′ end of XistAR RNA by 3′ RACE, but were unsuccessful, presumably due to the absence of a poly-A+ tail at the 3′ end of XistAR. In agreement, neither 5′ RACE nor RT–PCR with poly-A+ selected RNAs amplified XistAR. We therefore mapped the 3′ end of XistAR by overlapping RT–PCRs with total RNAs followed by SNP profiling (Fig. 3c–f). The 3′ end maps to ∼bp 13 at the very 5′ end of Xist. We independently delineated *XistAR* structure by RNA FISH using overlapping strand-specific probes (Fig. 4). Multiple RT–PCR amplicons and RNA FISH probes >5 kb upstream of Xist did not detect inactive X-specific transcription (Figs 3f and 4). The full-length XistAR RNA sequence is ∼2.8 kb in length and is predicted to be non-protein coding through the Coding Potential Calculator algorithm^29^.

**Figure 5:**
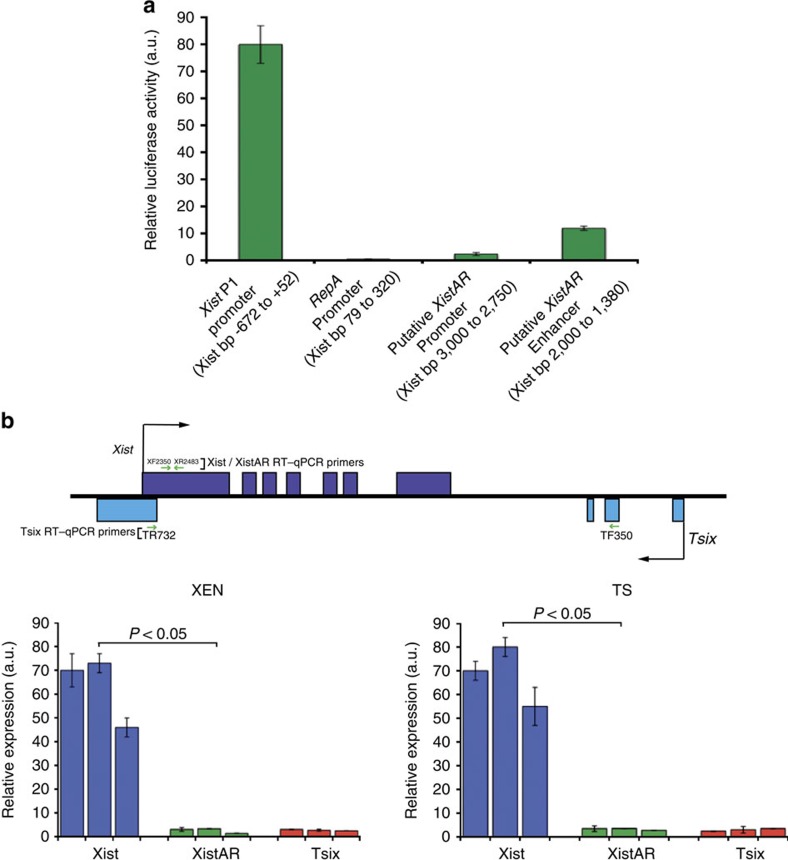
Identification of putative *XistAR* promoter and enhancer element. (**a**) Relative luciferase activity in cells transfected with constructs containing the *Xist* P1 promoter, *RepA* promoter, putative *XistAR* promoter and enhancer sequences. In the enhancer assay, the fragment was cloned upstream of the putative *XistAR* promoter sequence (see Methods for full details). Each construct was tested in triplicate in each of three separate transfections. a.u., arbitrary units. (**b**) Comparisons of relative expression of Xist, XistAR, Tsix RNAs by RT-qPCR in three XEN and TS cell lines (XistAR was profiled in *X*^ΔTsix^*X* cell lines). Each cell line was tested in three technical replicates. *P* values were calculated using Welch’s two-sample *t*-test. Error bars represent s.d.

Next, we mapped sequences in Xist exon 1 that may function as a promoter and enhancer of XistAR via luciferase reporter assays. In comparison to the Xist P1 promoter sequence^30^,^31^, we detected weak promoter activity in the fragment encompassing the 5′ end of XistAR and extending ∼200 bp upstream (bp 3,000–2,750 of Xist, in the same 5′-to-3′ orientation as the XistAR transcript) (Fig. 5a). A larger fragment extending to Xist bp 3,500 did not display stronger promoter activity. Although weaker than the Xist P1 promoter, the putative *XistAR* promoter sequence was stronger than the promoter of the *RepA* gene. Like XistAR, RepA is contained wholly within the first Xist exon; but, as opposed to the antisense orientation of XistAR, RepA is transcribed in the same orientation as Xist. Furthermore, scanning of several sequence fragments for enhancer activity in luciferase reporter assays identified bp 2,000–1380 as a putative XistAR enhancer (Fig. 5a). Nevertheless, the combined activity of these putative promoter and enhancer sequences was much weaker than the Xist P1 promoter. In agreement with the relatively weak activities of these sequences, RT–quantitative PCR (RT–qPCR) demonstrated that the XistAR transcripts are proportionately less abundant compared with Xist RNA, but similar to Tsix RNA expression (Fig. 5b and Supplementary Fig. 1). Also consistent with the reporter assays, the putative XistAR regulatory sequences display DNAseI hypersensitivity and histone H3-K4me3 enrichment in mouse cells in the Ensembl genome browser^32^,^33^.

### Functional characterization of *XistAR*

**Figure 6:**
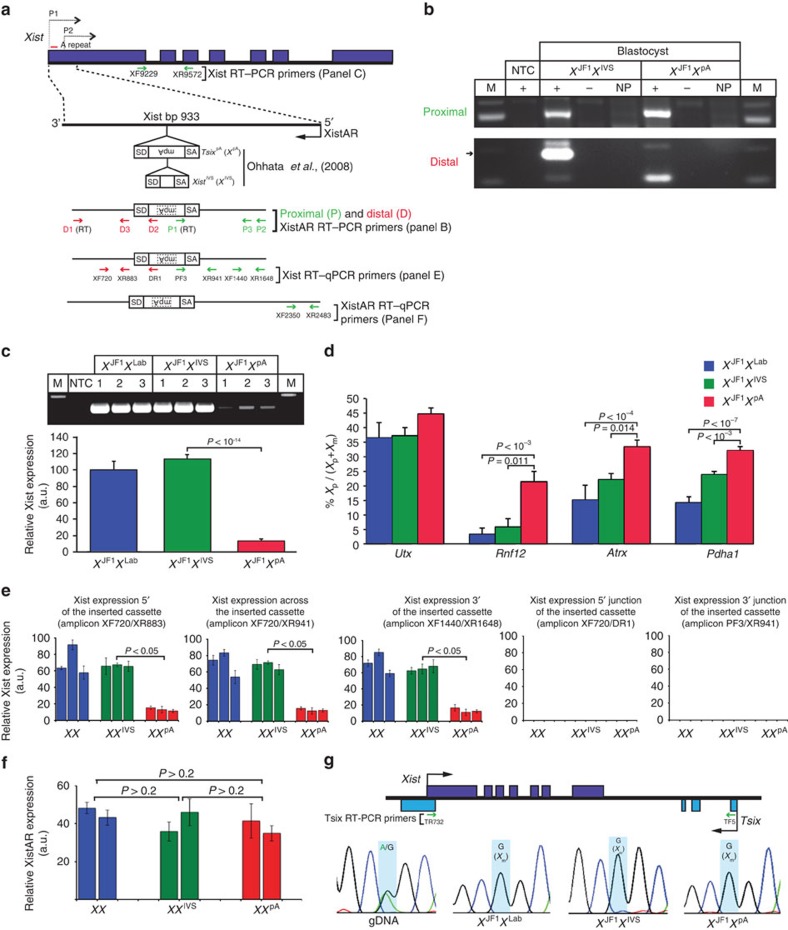
XistAR is required for robust Xist induction. (**a**) Schematic representation of *Tsix*^pA^ (*X*^pA^) and *Xist*^IVS^ (*X*^IVS^) mutations and the locations of the RT–PCR primers used. (**b**) RT–PCR detection of XistAR proximally and distally to the insertion cassette in *X*^JF1^*X*^IVS^ and *X*^JF1^*X*^pA^ blastocysts. M, marker; NTC, no template control; +, reaction with reverse transcriptase (RT); −, reaction without RT; NP, ‘no primer’ control RT reaction without added primer in the RT step but with primers included in the PCR step. (**c**) RT–PCR detection of Xist in *X*^JF1^*X*^Lab^, *X*^JF1^*X*^IVS^ and *X*^JF1^*X*^pA^ blastocysts. Xist levels were quantified via pyrosequencing (see Methods). Three blastocysts of each genotype were analysed. (**d**) Defective silencing of the X-linked genes *Utx*, *Rnf12*, *Atrx* and *Pdha1* in *X*^JF1^*X*^pA^ compared with *X*^JF1^*X*^Lab^ and *X*^JF1^*X*^IVS^ blastocysts. *X*_p_, paternal allele; *X*_m_, maternal allele. Allele-specific expression levels in **c**,**d** were quantified by pyrosequencing and compared using Welch’s two-sample *t*-test. In **d** *P* values were adjusted using the Bonferroni correction to account for multiple testing. Five to fifteen embryos were analysed for each gene. Error bars represent s.d. (**e**) RT–qPCR comparisons of Xist expression proximally, across, distally and at the junctions of the inserted cassette in *XX*, *XX*^IVS^ and *XX*^pA^ blastocysts. Data were normalized to Gapdh expression. Three different embryos were analysed from each genotype in triplicate. a.u., arbitrary units. (**f**) Relative quantification of XistAR expression by RT–qPCR upstream of the inserted cassette (that is, towards the 5′ end of XistAR) in *XX*^Lab^, *XX*^IVS^ and *XX*^pA^ blastocysts. The amplicon is unique to XistAR and not present in mature Tsix (location of primers shown in Fig. 6a). Two different embryos of each genotype were analysed in triplicate. In **e**,**f** *P* values were calculated using Welch’s two-sample *t*-tests. Error bars represent s.d. (**g**) Allele-specific analysis of Tsix expression in *X*^JF1^*X*^Lab^, *X*^JF1^*X*^IVS^ and *X*^JF1^*X*^pA^ blastocysts. The SNP position, at 110 bp of Tsix, is shaded in blue. Representative genomic DNA displays both alleles.

To define the functional importance of XistAR, we employed a mouse strain in which antisense but not sense transcription within Xist exon 1 is disrupted via the insertion of a human γ-globin intronic cassette harbouring multiple polyadenylation sequence (mpA) in the antisense orientation at bp 933 of Xist (*Tsix*^pA^; *X*^pA^) (ref. 34) (Fig. 6a). As a control, we used a strain that contained the same intronic cassette but without the mpA sequence (*Xist*^IVS^; *X*^IVS^) (refs 34, 35) (Fig. 6a). Both strains were originally generated to examine *Tsix* function. The presence of the mpA sequence truncates antisense expression in *Tsix*^pA^; its absence leaves the *Xist*^IVS^ allele relatively unaffected. Importantly, sense transcription can still occur due to splicing donor and acceptor sequences that flank the inserted cassette in the sense orientation in both the *Tsix*^pA^ and *Xist*^IVS^ alleles. We first examined E3.5 blastocyst-stage embryos, since *XX*^pA^ embryos display lethality shortly thereafter during gestation^34^. Consistent with truncation of antisense transcription, we found that XistAR is detected proximally but not distally with respect to the inserted cassette in *XX*^pA^ blastocysts by RT–PCR (Fig. 6b). In contrast, *XX*^IVS^ blastocysts displayed expression both proximally and distally to the cassette (Fig. 6b). We next tested expression of Xist by RT–PCR in F1 hybrid embryos and found that it was markedly decreased in *X*^JF1^*X*^pA^ but not in *X*^JF1^*X*^IVS^ compared with *X*^JF1^*X*^Lab^ blastocysts (Fig. 6c). The Xist amplicon spans exons 1–3, and thus detects Xist isoforms generated from both Xist P1 and P2 promoters^31^.

Next, we quantified the allele-specific expression of Xist as well as of four genes distributed across the X chromosome, *Utx*, *Rnf12, Atrx* and *Pdha1*, by pyrosequencing. While *Rnf12, Atrx* and *Pdha1* are subject to X inactivation, *Utx* escapes X inactivation^23^,^36^,^37^. Since blastocysts undergo imprinted inactivation of the paternal X in all cells, we generated WT and mutant F1 hybrid embryos to exploit expressed SNPs to quantify the level of homologue-specific expression of the X-linked genes. Xist was only expressed at ∼13% of the level in *X*^JF1^*X*^pA^ compared with control *X*^JF1^*X*^IVS^ and *X*^JF1^*X*^Lab^ blastocysts (Fig. 6c). The decrease in Xist RNA from the paternal X in *X*^JF1^*X*^pA^ embryos coincided with an increase in expression of *Rnf12*, *Atrx* and *Pdha1* from that X chromosome (Fig. 6d). *Utx* also displayed a relative increase in expression from the paternal X, suggesting it too is derepressed from the inactive X in *X*^JF1^*X*^pA^ embryos (Fig. 6d). The increase in paternal allele expression of all four genes in the *X*^JF1^*X*^IVS^ relative to *X*^JF1^*X*^Lab^ embryos is postulated to be due to defective Xist RNA function from the *Xist*^IVS^ allele as a result of a residual 16-bp sequence in Xist left behind after the inserted cassette is spliced^35^.

XistAR could function to induce Xist RNA expression. Alternatively, XistAR could influence Xist RNA elongation. To distinguish amongst these two possibilities, we compared Xist expression upstream and downstream of the inserted cassette in *XX*^pA^ and *XX*^IVS^ blastocysts by RT–qPCR. We found that Xist expression was similarly reduced on either side of the inserted cassette in *XX*^pA^ embryos (Fig. 6e). By contrast, *XX*^IVS^ embryos showed similar Xist levels to that of WT embryos. An Xist amplicon bridging sequences on either side of the inserted cassette recapitulated the expression pattern found on either side of the cassette in both genotypes. We also validated that the inserted cassette was equally and efficiently spliced to form mature Xist RNA in *XX*^pA^ and *XX*^IVS^ embryos (Fig. 6e). These results suggest a role for XistAR expression in driving or enhancing Xist expression rather than in mediating Xist elongation.

We also examined if the absence of a poly-A+ tail in XistAR contributed to its instability, thus leading to the low steady-state levels of XistAR. The *Tsix*^pA^ allele offered a venue to address this possibility, since a poly-A+ sequence is added to XistAR in the *Tsix*^pA^ mutation (Fig. 6a). On assaying XistAR expression upstream of the inserted cassette (that is, towards the 5′ end of *XistAR*) by RT–qPCR, we found that the *XX*^pA^ blastocysts did not exhibit a significant increase in XistAR abundance compared with *XX*^IVS^ and *XX* embryos (Fig. 6f). The inclusion of a poly-A+ tail, therefore, did not appear to increase the stability of the XistAR transcript (Fig. 6f). Thus, the absence of a poly-A+ tail may not be a contributing factor in the low XistAR levels.

We also tested if Tsix is ectopically expressed from the paternal X in *X*^JF1^*X*^pA^ blastocysts, as would be expected if Xist RNA is insufficiently induced^38^. RT–PCR followed by Sanger sequencing demonstrated that Tsix is expressed only from the maternal X in *X*^JF1^*X*^pA^ blastocysts, similarly to *X*^JF1^*X*^IVS^ and *X*^JF1^*X*^Lab^ embryos (Fig. 6g). Thus, the residual Xist expression from the paternal *X*^pA^ X chromosome appears sufficient to repress Tsix in *cis*.

**Figure 7:**
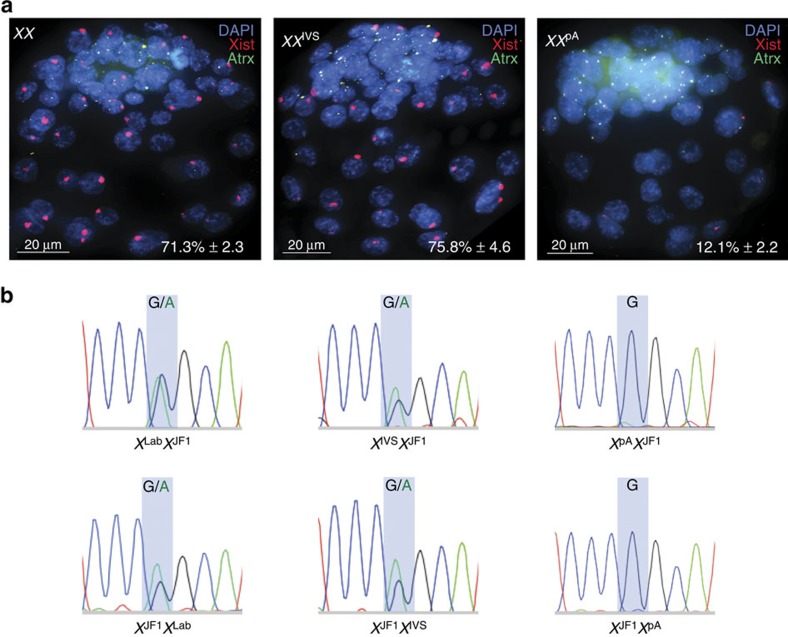
Profiling of Xist RNA coating in *XX*^Lab^, *XX*^IVS^ and *XX*^pA^ embryos. (**a**) RNA FISH detection of Xist RNA coating in *XX*^Lab^, *XX*^IVS^ and *XX*^pA^ blastocysts. Red, XistAR RNA; green, Xist RNA; white, Atrx; blue, nucleus. Atrx RNA marks the active X chromosome. Numerical values in the images indicate the percentage of nuclei that display Xist RNA coats; ±, s.d. *n*=3 embryos per genotype. *XX*^pA^ embryos display significantly fewer nuclei with Xist RNA coating compared with both *XX*^IVS^ (*P*<10^−3^; Welch’s two-sample *t*-test) and *XX*^Lab^ (*P*<10^−4^). (**b**) Sanger sequencing chromatograms of *Xist* cDNAs in F1 hybrid *X*^JF1^*X*^Lab^, *X*^JF1^*X*^IVS^ and *X*^JF1^*X*^pA^ E6.5 epiblasts (top). Similar analysis was performed with embryonic epiblasts from the reciprocal cross of these genotypes (bottom). In *X*^JF1^*X*^pA^ and *X*^pA^*X*^JF1^ epiblasts, Xist is expressed exclusively from the *X*^JF1^X chromosome.

We next assayed Xist expression in blastocysts of all three genotypes by RNA FISH using a probe that is expected to detect Xist transcripts from both P1 and P2 promoters (Fig. 1a). We observed a marked decrease in Xist RNA coating in *XX*^pA^ but not in *XX*^IVS^ compared with WT *XX* blastocysts (Fig. 7a). Moreover, the Xist RNA:X-chromosomal associations in *XX*^pA^ embryos were qualitatively smaller than in *XX*^IVS^ and *XX*^Lab^ blastocysts. Together, the results presented in Figs 6 and 7 indicate that the truncation of XistAR results in a significant reduction in Xist RNA expression and coating and an increase in the expression of genes that are normally X inactivated.

Finally, we tested if the *Tsix*^pA^ mutation alters Xist induction during random X inactivation, as it does in imprinted X inactivation. The epiblast lineage normally undergoes random X inactivation^25^,^39^,^40^,^41^, we therefore assayed Xist expression in epiblasts of F1 hybrid mutant and WT embryos. In agreement with the failure of Xist induction from the *X*^pA^ paternal X chromosome in imprinted X inactivation, *Tsix*^pA^-heterozygous epiblasts displayed Xist induction exclusively from the WT *X*^JF1^ X chromosome (Fig. 7b). By contrast, *Xist*^IVS^ and WT heterozygous epiblasts expressed Xist from both Xs, consistent with random X inactivation (Fig. 7b).

## Discussion

In summary, we have identified and functionally characterized a novel Xist antisense lncRNA, XistAR, that is expressed only from the inactive X chromosome and which we propose is required for robust expression of Xist RNA. An intriguing possibility is that XistAR functions as an enhancer RNA (eRNA) to induce Xist. eRNAs are broadly transcribed from enhancers and may contribute to their function (reviewed in ref. 42). Like XistAR, eRNAs exhibit a 5′ cap are often not spliced or polyadenylated, and have short half-lives compared with mRNAs^42^. To enhance Xist expression, the transcription of XistAR, rather than the RNA itself, may be sufficient. Since XistAR is transcribed close to the 5′ end of Xist, the act of its transcription may remodel the shared chromatin and mark it as transcriptionally competent, thus facilitating Xist expression. In agreement, in the *Tsix*^pA^ mutants XistAR transcription does not reach the 5′ end of Xist and Xist expression is reduced by ∼90%. Moreover, that in the *Xist*^IVS^ mutant blastocysts Xist expression does not decrease despite the insertion of >1 kb sequence also supports a functional role for antisense transcription and not the XistAR RNA itself in Xist induction. Nevertheless, we cannot rule out a role for the XistAR RNA in enhancing Xist expression.

In *Tsix*^pA^, instead of the truncation of XistAR, the added mpA sequence or the larger size of the inserted cassette could formally be responsible for the reduced Xist expression. For example, the larger insertion could impede transcription in the vicinity of the cassette. That Xist expression from both the P1 and P2 promoters is reduced but XistAR expression upstream of the inserted cassette remains unaffected in *XX*^pA^ compared with *XX*^IVS^ blastocysts (Fig. 6f), however, argues against transcriptional interference by the cassette as the proximate cause of diminished Xist expression from the *X*^pA^ X chromosome.

The 3′ end of XistAR lacks a poly-A+ tail but resides in the region of Xist termed the ‘A-repeat’, which can adapt thermodynamically stable stem–loop secondary RNA structures in solution^43^,^44^. The reverse complement of the A-repeat antisense sequence found in XistAR should also be able to base pair to form similar stem–loop structures, perhaps conferring stability to the transcript normally provided by a poly-A+ tail. Unexpectedly, the inclusion of a poly-A+ tail in the *Tsix*^pA^ allele did not increase XistAR abundance relative to *Xist*^IVS^ and WT alleles. The results therefore favour the notion that the low steady-state levels of XistAR are due to weak induction and/or inherently short half-life rather than instability due to the lack of a poly-A+ tail.

In both mice and humans, Xist transcripts can initiate from two separate start sites. Each transcript is believed to be driven by its own promoter, P1 and P2. P2 transcript starts 1,503 bp downstream of the P1-driven transcript^31^. XistAR appears to influence the induction of both Xist transcripts. An amplicon downstream of P2 designed to detect both Xist isoforms by RT–PCR demonstrated significantly reduced Xist expression in *XX*^pA^ embryos (Fig. 6c). Similarly, an Xist RNA FISH probe generated from Xist exon 7 template that can detect both transcripts shows significantly fewer nuclei with Xist RNA coating in *XX*^pA^ embryos (Fig. 7a). Since in *Tsix*^pA^ the γ-globin intronic cassette is inserted at Xist bp 933, a truncated XistAR transcript that traverses the putative P2 promoter in *XX*^pA^ embryos is nevertheless incapable of inducing the P2 Xist RNA. Thus, the complete transcription of XistAR appears necessary to induce both Xist P1 and P2 isoforms.

Intriguingly, Tsix and XistAR have opposing effects on Xist expression, despite both being transcribed in the antisense orientation to Xist. Whereas Tsix inhibits Xist expression^45^,^46^,^47^, XistAR appears to induce Xist. The divergent effects of the two RNAs may be explained by differences in the 3′ ends of the two transcripts. Tsix is transcribed across the very 5′ end of Xist and through the Xist promoter^47^,^48^, whereas XistAR transcript ends just shy of the 5′ end. Inhibition of Xist requires that antisense transcription extends across the Xist P1 promoter^34^. As a result, Tsix but not XistAR may be capable of suppressing Xist expression.

Both Xist and XistAR traverse the A-repeat sequence at the 5′ end of Xist. The A-repeat is believed to be important for Xist RNA induction and/or its gene-silencing function^38^,^44^,^49^. In addition to Xist, the A-repeat sequence is proposed to be a part of the 1.6-kb RepA transcript that is expressed from within exon 1 of *Xist* and in the same 5′-to-3′ orientation as the Xist RNA^49^. RepA is believed to activate Xist by recruiting polycomb repressive complex 2 (PRC2) to the Xist locus^49^. PRC2 catalyses trimethylation of lysine 27 of histone H3 (H3K27me3) and triggers a transient heterochromatic state within the 5′ end of Xist^12^,^49^. This 5′ Xist sequence overlaps with both RepA and XistAR. It is therefore possible that a perturbation of the RepA RNA by the inserted human γ-globin intronic cassette is the cause of Xist RNA diminishment in *XX*^pA^ embryos. However, that Xist is able to be upregulated in the control *XX*^IVS^ embryos argues against this notion. We instead propose that the function of the A-repeat in inducing Xist is partly or wholly executed via the antisense XistAR transcript. Notably, antisense transcription in Xist exon 1 from the inactive X has also recently been documented in human cells^50^. An earlier study in mice may also have identified an Xist antisense transcript originating from the inactive X in the *XistAR* interval, as the antisense transcript was amplified in female but not male embryonic stem cells^51^.

In addition to PRC2-catalysed chromatin modification, Xist activation is believed to be controlled by the pluripotency-associated factors such as NANOG and OCT4 (refs 8, 10). Recently, the histone H4 lysine 16 acetyltransferase (H4-K16ac) MOF^52^,^53^,^54^ has been shown to control the pluripotency network and to control Xist expression either through the pluripotency proteins or through Tsix^52^. Intriguingly, MOF binds the *XistAR* DNA sequence and acts as a negative regulator of Xist^52^,^54^. Depletion of MOF results in Xist induction from the active X in male embryonic stem cells^52^,^54^, potentially via derepression of XistAR. Thus, an attractive hypothesis is that counterbalancing functions of MOF and PRC2 control Xist induction via XistAR. Whereas H4-K16ac may inhibit XistAR expression, PRC2-catalysed H3-K27me3 at the 5′ end of Xist induces XistAR and thus Xist. Future work will define precisely how XistAR is induced or silenced and how XistAR influences Xist expression.

## Methods

### Ethics statement

This study was performed in strict accordance with the recommendations in the Guide for the Care and Use of Laboratory Animals of the National Institutes of Health. All animals were handled according to the protocols approved by the University Committee on Use and Care of Animals at the University of Michigan (Protocol #PRO00004007).

### Mice

The generation and characterization of *Tsix*^pA^ and *Xist*^IVS^ strains has been described previously^34^. Both strains were maintained on the Bl/6 background by breeding heterozygous *Tsix*^pA^ and *Xist*^IVS^ females with C57Bl/6 males. The X-linked *GFP* transgenic (*X-GFP*) and JF1 strains have also been described previously^15^,^36^,^55^,^56^.

### Embryo dissections and processing

E3.5 embryos were isolated essentially as described^19^. Embryos were flushed from the uterine limbs in 1 × PBS (Invitrogen, #14200075) containing 6 mg ml^−1^ BSA (Invitrogen, #15260037). Zona pellucida surrounding E3.5 embryos were removed through incubation in cold acidic Tyrode’s solution (Sigma, #T1788), followed by neutralization through several transfers of cold M2 medium (Sigma, #M7167). Green fluorescent protein (GFP) fluorescence conferred by the paternal transmission of the *X-GFP* transgene was used to distinguish female from male embryos, since only females inherit the paternal X chromosome. Embryos were either lysed for RNA isolation or plated onto gelatin-coated glass coverslips in 1 × PBS with 6 mg ml^−1^ BSA for RNA FISH staining. Excess solution was aspirated, and the plated embryos were air dried for 15 min. After drying, embryos were permeabilized and fixed in 50 μl solution of 0.05% Tergitol (Sigma, #NP407) and 1% paraformaldehyde (Electron Microscopy Sciences, # 15710) in 1 × PBS for 10 min. Excess solution was tapped off, and coverslips were rinsed thrice with 70% ethanol and stored in 70% ethanol at −20 °C before RNA FISH.

For isolation of E6.5 embryos, individual implantation sites were cut from the uterine limbs and decidua were removed with forceps in 1 × PBS/6 mg ml^−1^ BSA. Embryos were dissected from the decidua, and the Reichert’s membranes surrounding post-implantation embryos were removed using fine forceps. For separation of extra-embryonic and epiblast portions of E6.5 embryos, fine forceps were used to physically bisect the embryos at the junction of the epiblast and extra-embryonic ectoderm. The epiblast was further distinguished by GFP fluorescence conferred by the paternally transmitted *X-GFP* transgene; the transgene is mosaically expressed in the epiblast due to random X inactivation but is silenced in the extra-embryonic tissues because of imprinted X inactivation of the paternal X^15^,^36^,^55^,^56^. Extra-embryonic and embryonic epiblast cells were then separately plated in 0.25 × PBS with 6 mg ml^−1^ BSA onto gelatinized coverslips. The samples were permeabilized, fixed and stored in 70% ethanol as described above for E3.5 embryos, before RNA FISH.

### Derivation and culture of TS cell lines

TS cell derivation, culture and characterization was carried out essentially as described previously^14^,^15^,^18^. E3.5 embryos were flushed from the uterus in MEMα (Invitrogen, #12561) with 10% fetal bovine serum (FBS; Invitrogen, #10439-024) and plated individually on mouse embryonic fibroblast (MEF) in wells of a 96-well tissue culture dish with medium consisting of RPMI (Invitrogen, #21870076), 20% FBS, 1 mM sodium pyruvate (Invitrogen, #11360-070), 100 μM β-mercaptoethanol (Sigma, #M7522), 2 mM L-glutamine (Invitrogen, #25030), 37.5 ng ml^−1^ FGF4 (R&D Systems, #235-F4-025) and 1.5 μg ml^−1^ heparin (Sigma, #H3149-10KU). Following 5 days of growth at 37 °C with 5% CO_2_, blastocyst outgrowths were dissociated with 0.05% trypsin (Invitrogen, #25300-054). Dissociated cells were plated on MEFs and cultured at 37 °C with 5% CO_2_. Once TS lines were established, TS cells were collected for total RNA preparation using Trizol (Invitrogen, #15596-018). For RNA FISH, TS cells were split onto gelatin-coated glass coverslips and allowed to grow for 2–3 days. The cells were then permeabilized through sequential treatment with ice-cold cytoskeletal extraction (CSK) buffer (100 mM NaCl, 300 mM sucrose, 3 mM MgCl_2_ and 10 mM PIPES buffer, pH 6.8) for 30 s, ice-cold CSK buffer containing 0.4% Triton X-100 (Fisher Scientific, #EP151) for 30 s, followed twice with ice-cold CSK buffer for 30 s each. After permeabilization, cells were fixed in 3% paraformaldehyde for 10 min. Cells were then rinsed thrice in 70% ethanol and stored in 70% ethanol at −20 °C before RNA FISH.

### Derivation and culture XEN cell lines

XEN cells were derived, cultured and characterized as described previously^15^,^16^. E3.5 embryos were flushed from the uterus with MEMα/10% FBS and plated individually on MEFs in wells of a 96-well tissue culture dish with 750 μl of XEN-derivation media (MEMα, 50 μg ml^−1^ penicillin/streptomycin (Invitrogen, # 15070063), 20% FBS, 1 mM sodium pyruvate, 100 μM β-mercaptoethanol, 2 mM L-glutamine, 100 μM nonessential amino acids (GIBCO, #11140-050), 1,000 units ml^−1^ leukemia inhibitory factor (Millipore # ESG1107). Following 6–8 days of growth at 37 °C with 5% CO_2_, blastocyst outgrowths were dissociated with 0.05% trypsin. Dissociated cells were plated into individual wells of a 96-well dish containing MEFs and cultured at 37 °C with 5% CO_2_. The cells were then split into a single well of a four-well dish containing MEFs. After confluency, the cells were trypsinized and plated into a gelatinized well of a six-well dish in XEN growth media (MEMα, 20% FBS, 1 mM sodium pyruvate, 100 μM β-mercaptoethanol, 2 mM L-glutamine and 100 μM nonessential amino acids). Total RNA was collected from XEN cells using Trizol for RT–PCR. For RNA FISH, XEN cells were split onto gelatin-coated glass coverslips and allowed to grow for 2–3 days. The cells were then permeabilized, fixed and stored in 70% ethanol as described above for TS cells.

### Derivation and culture of EpiSC lines

EpiSC lines were derived, cultured and characterized as described^21^,^22^,^23^,^57^. Briefly, individual embryos were plated on quiescent MEF feeder cells in K15F5 medium containing knockout DMEM (GIBCO, #10829-018) supplemented with 15% knockout serum replacement (GIBCO, #A1099201), 5% FBS (GIBCO, #104390924), 2 mM L-glutamine, 1 × nonessential amino acids and 100 μM β-mercaptoethanol. After 5–6 days, outgrowths were partially dissociated with 0.05% trypsin. The partial dissociates were plated individually on MEFs in a well of a four-well dish and cultured for an additional 4–6 days in K15F5 medium. The culture was then passaged by a brief exposure (2–3 min) to 0.05% trypsin/EDTA (Invitrogen, # 25300-054) with gentle pipetting to prevent complete single-cell dissociation of pluripotent clusters, and plated on MEFs in a single well of a six-well dish in K15F5 medium. Morphologically distinct EpiSC colonies became evident over the next 4–8 days. EpiSC colonies were manually dissociated into small clusters using a glass needle and plated on MEFs in a single well of a four-well dish in EpiSC medium consisting of knockout DMEM supplemented with 20% knockout serum replacement, 2 mM Glutamax (GIBCO, #35050061), 1 × nonessential amino acids, 100 μM β-mercaptoethanol and 10 ng ml^−1^ FGF2 (R&D Systems, #233-FB).

### Isolation of total RNA and mRNA from cells

Total RNA was purified from cultured TS, XEN and EpiSC cell lines using Trizol reagent (Life Technologies, #15596018). Total RNA was treated with DNase (Life Technologies, #AM1906) to remove any contaminant genomic DNA. mRNA was isolated from DNase-treated total RNA using Dynabeads mRNA DIRECT Micro Kit (Life Technologies, #61012).

### Isolation of total RNA and mRNA from embryos

Total RNA from E3.5 embryos was purified by lysis in 10 μl extraction buffer of the PicoPure RNA Isolation Kit, followed by the manufacturer’s instructions (Life Technologies #KIT0204). Purified total RNA was resuspended in 30 μl of elution buffer. mRNA from E3.5 embryos was purified by lysis in 100 μl lysis/binding buffer of the Dynabeads mRNA DIRECT Kit, followed by the manufacturer’s instructions. Purified mRNA was resuspended in 50 μl of elution buffer.

### Reverse transcription–PCR

To exclude the RT and amplification of the sense Xist RNA by cell intrinsic primers^20^, a modified RT–PCR protocol was implemented to generate and specifically amplify XistAR. A unit of 1 μg DNase-treated total RNA was reverse transcribed (SuperScript III First Strand Synthesis System, Invitrogen # 18080-051) with Abridged Universal Amplification Primer (AUAP) (adapted from Invitrogen 5′ RACE System, # 18374-058)-tagged strand-specific RT primers (see Supplementary Table 1 for list of primers) followed by RNase H (Invitrogen #18021-071) treatment to degrade the RNA. The RT reaction mixture was then heat denatured at 95 °C for 5 min followed by snap cooling on ice. The heat-denatured product was then purified thrice by NucleoSpin Gel and PCR Clean-up kit (Clontech #740609.250), to ensure the complete removal of any RT primer. Residual RT primer could serve in the PCR step to amplify any cDNAs spuriously generated by RT of sense-strand Xist RNA by cell intrinsic primers^20^. A portion of the cDNA was then PCR amplified using AUAP and a gene-specific reverse primer (Supplementary Table 1). The PCR product was then subjected to nested PCR with AUAP and an internal gene-specific primer. Nested PCR products were run on a 1.5% agarose gel and the eluted cDNA was Sanger sequenced.

For the proximal and distal RT–PCRs from *Tsix*^pA^ and *Xist*^IVS^ blastocysts in Fig. 6, DNase-treated total RNA from E3.5 female embryos was used as template. To increase the specificity of amplification of the antisense transcript, the RT primer in the proximal amplicon maps to the inserted cassette in both *Tsix*^pA^ and *Xist*^IVS^ mutations. Similarly, in the distal amplicon the reverse primer in the initial round of PCR resides in the inserted cassette. The RT primers were AUAP coupled to provide further specificity during PCR amplification; AUAP was then employed in the initial and nested PCR steps.

Allele-specific expression of Tsix in *X*^JF1^*X*^Lab^, *X*^JF1^*X*^IVS^ and *X*^JF1^*X*^pA^ blastocysts was assessed by RT–PCR using Invitrogen SuperscriptIII one-step RT–PCR system (Invitrogen, #12574-026) using the primer pair TF5 and TR732 (Supplementary Table 1) and followed by Sanger sequencing. The Tsix amplicon spanned exon 1 to exon 4 of Tsix with the SNP position at 110 bp of Tsix.

### 5′ rapid amplification of cDNA ends

The 5′ end of XistAR was mapped using First Choice RLM-RACE kit (Ambion, catalogue #AM1700) with some modifications. Briefly, 5′ RLM-RACE exploits the ^7m^G cap at the 5′ end of RNA polymerase II-transcribed transcripts to precisely map the 5′ end of RNAs^27^,^28^. An RNA adapter is ligated only to RNAs that have an intact ^7m^G cap. These adapter-ligated RNAs together with a primer comprised of the unique adapter sequence and a gene-specific primer facilitates mapping of the very 5′ end of the target RNAs by RT–PCR. The RNA adapter is not ligated to fragmented or degraded RNAs because the 5′ PO_4_ in these RNAs is dephosphorylated before adapter ligation, thus preventing their amplification by RT–PCR. As a result, only RNAs with an intact 5′ end are amplified by RT–PCR.

A unit of 10 μg of total RNA was treated with calf intestine alkaline phosphatase (CIP) to remove free 5′ phosphates from molecules such as ribosomal RNA, fragmented mRNA, tRNA and contaminating genomic DNA. The cap structure found on intact 5′ ends of transcript is not affected by calf intestine alkaline phosphatase. The RNA was then treated with tobacco acid pyrophosphatase to remove the cap structure from full-length transcript, leaving a 5′ monophosphate. A 45 base RNA adapter oligonucleotide (provided with the kit) was then ligated to the RNA population using T4 RNA ligase and primed with oligo XF1851 (Supplementary Table 1) for cDNA synthesis. Adapter-specific primers and transcript-specific oligos XF2121 and XF2296 were used for amplification of the specific RACE product in two sequential PCR reactions, respectively.

### Quantification of allele-specific expression

Allele-specific expression was quantified using Qiagen PyroMark sequencing platform. Amplicons containing SNPs were designed using the PyroMark Assay Design software. cDNAs were synthesized using Invitrogen SuperScript III One-Step RT–PCR System (Invitrogen, #12574-026). Following the PCR reaction, 5 μl of a total of 25 μl was run on a 3% agarose gel to assess the efficacy of the RT and amplification. The samples were then prepared for pyrosequencing according to the standard recommendations for use with the PyroMark Q96 ID sequencer.

To quantify the relative expression of Xist RNA between embryos of different genotypes, mRNA was isolated from individual E3.5 F1 hybrid WT (derived from a cross of *Mus domesticus*-derived Bl/6 female to *M. molossinus*-derived *X*^JF1^ male (F1_i_), as well as from the reciprocal cross (F1_r_)), F1_r_ *Xist*^IVS^ (JF1 female × *Xist*^IVS^*Y* male (on a Bl/6 background)), F1_r_ *Tsix*^pA^ (JF1 female x *Tsix*^pA^*Y* male (also on a Bl/6 background)) embryos. Sex of the embryos was then assayed by amplifying Xist RNA by RT–PCR and profiling a SNP in the amplicon to determine if the cDNA maps to the paternal X; and, by examining if the X-linked *Pdha1* gene is biallelically detected by RT–PCR and Sanger sequencing. mRNAs from each of five WT F1_i_ fully grown blastocysts, in which Xist RNA is derived solely from the *X*^JF1^ paternal X, was individually mixed with an equivalent amount of mRNA from each of six fully grown blastocysts from each of three different F1_r_ genotypes, WT, *Xist*^IVS^ or *Tsix*^pA^, all of which express Xist only from the *M. domesticus*-derived paternal X. RT–PCR was then performed with 2 μl of the mixed mRNAs using the Invitrogen SuperScript III One-Step RT–PCR System. In WT F1_i_ blastocyst embryos, Xist is only expressed from the paternal *X*^JF1^ allele due to imprinted X inactivation. In the *Xist*^IVS^ and *Tsix*^pA^ mutant F1_r_ blastocysts, on the other hand, the only transcriptionally competent Xist allele is *M. domesticus* derived. In the cDNAs generated from an equal mixture of F1_i_ WT with F1_r_ *Xist*^IVS^ or *Tsix*^pA^ mutant female mRNAs, a SNP in *Xist* was exploited to distinguish and quantify the relative expression of Xist RNAs in *Xist*^IVS^ or *Tsix*^pA^ compared with WT embryos by pyrosequencing. In the mRNA mixtures, the F1_i_ WT Xist RNA originates from the *M. molossinus*-derived *X*^JF1^; in the F1_r_ *Xist*^IVS^ or *Tsix*^pA^ mutants, the expressed *Xist* allele is *M. domesticus* derived. Expression of *M*. *molossinus* and the *M. domesticus* Xist alleles was normalized by comparing Xist expression in WT F1_i_ and F1_r_ blastocysts by pyrosequencing. In WT F1_i_ and F1_r_ blastocysts, Xist expression was undetectable from the maternal X chromosome by pyrosequencing.

The allele-specific expression of the X-linked genes *Utx*, *Rnf12*, *Atrx* and *Pdha1* was also analysed by pyrosequencing in individual blastocysts, but without mixing mRNAs from WT and mutant strains as for Xist above. All amplicons, including Xist, spanned at least one intron, thus excluding any amplified contaminating genomic DNA sequence due to size differences. Control reactions lacking reverse transcriptase for each sample were also performed to rule out genomic DNA contamination. Five to fifteen biological replicates were performed for each gene. Gene expression was compared between genotypes using pairwise *t*-tests. *P* values were adjusted using the Bonferroni correction to account for multiple testing.

### RT–qPCR

RT–qPCR was performed using SYBR Green-based (Kapa Biosystem #KK4650) relative quantification method on an Eppendorf Realplex Mastercycler. *Gapdh* was used as the housekeeping internal control for data normalization. Total RNA and mRNA were DNaseI treated before RT. cDNA was synthesized using SuperScript III First Strand Synthesis System (Invitrogen #18080-051), following the manufacturer’s instructions. Control reactions lacking reverse transcriptase for each sample were also performed to rule out genomic DNA contamination. For amplification of Xist, XistAR and Tsix in Fig. 5b, the RT and PCR reactions were performed using the modifications as described above in the ‘Reverse transcription–PCR’ section to exclude any RT and amplification due to cell intrinsic primers. The efficiency of the primer pairs ranged between 95 and 106%. For Xist primer pair, the efficiency was 95%; for XistAR primer pair, 96%; and, for Tsix primer pair, 106%. RT and PCR primers used are listed in Supplementary Table 1. *P* values for all RT–qPCR results were calculated using Welch’s two-sample *t*-tests.

### RNA fluorescence *in situ* hybridization

RNA FISH probes from double-stranded DNA templates were created by randomly priming DNA using BioPrime DNA Labeling System (Invitrogen, #18094011). FISH probe templates and procedures have been described previously^15^,^36^,^56^. Briefly, probes were labelled with Fluorescein-12-dUTP (Invitrogen), Cy3-dCTP (GE Healthcare, #PA53021) or Cy5-dCTP (GE Healthcare, #PA55031). Labelled probes for multiple genes were precipitated in a 0.3-M sodium acetate (Teknova, #S0298) solution along with 300 μg of yeast tRNA (Invitrogen, #15401-029), 15 μg of mouse COT-1 DNA (Invitrogen, #18440-016) and 150 μg of sheared, boiled salmon sperm DNA (Invitrogen, #15632-011). The solution was then spun at 21,000*g* for 20 min at 4 °C. The resulting pellet was washed in 70% ethanol, then washed in 100% ethanol, dried and resuspended in deionized formamide (ISC Bioexpress, #0606-500 ML). The resuspended probe was denatured via incubation at 90 °C for 10 min followed by immediate 5 min incubation on ice. A 2 × hybridization solution consisting of 4 × SSC, 20% dextran sulfate (Millipore, #S4030) and 2.5 mg ml^−1^ purified BSA (New England Biolabs, #B9001S) was added to the denatured solution. The probe was then pre-annealed by incubation at 37 °C for 1 h to minimize probe hybridization to repetitive sequences. Probes were stored at −20 °C until use.

Strand-specific RNA FISH (ssRNA FISH) probes were labelled with Fluorescein-12-UTP (Roche, #11427857910) or Cy3-CTP (GE Healthcare, # 25801086) using the Invitrogen MAXIscript Kit (Invitrogen, #AM-1324). To detect XistAR, a DNA template spanning Xist bp 953–1,440 was transcribed to generate single-stranded RNA probe. A single-stranded probe detecting Xist RNA was generated using PCR amplified DNA from Xist exon 7. Labelled probes were column purified (Roche, #11814427001) and precipitated in a 0.25-M ammonium acetate solution essentially as described above for double-stranded RNA FISH probes, but without the addition of COT-1 DNA. Probes were resuspended also as described for double-stranded RNA FISH probes and stored at −20 without pre-annealing. Embryos and cells were hybridized to the probe overnight in a humid chamber at 37 °C. The samples were then washed thrice for 7 min each while shaking at 39 °C with 2 × SSC/50% formamide, twice with 2 × SSC and twice with 1 × SSC. A 1:250,000 dilution of 4,6-diamidino-2-phenylindole (Invitrogen, #D21490) was added to the third 2 × SSC wash. The embryos were then mounted in Vectashield (Vector Labs, #H-1200).

### Luciferase promoter and enhancer assays

The selected promoters and enhancer sequences analysed are listed in Table 1. Each fragment was PCR amplified and cloned into the basic pNL-vector (Promega) upstream of the NanoLuc luciferase reporter gene. For promoter assays, the fragments were cloned into KpnI/HindIII sites of the promoterless pNL plasmid. For enhancer assays, the fragments were first inserted upstream of the minimal promoter element into the KpnI/HindIII sites of the pNL-minP NanoLuc luciferase reporter plasmid (Promega). Subsequently, the fragment (Xist bp 2,000–1,380) that displayed the highest enhancer activity was cloned upstream of the putative *XistAR* promoter sequence (Xist bp 3,000–2,750). The plasmids were transfected into female XEN cells plated in a 96-well format. Each well was transfected with 100 ng of pNL-NanoLuc reporter construct and 6 ng of transfection control plasmid encoding the firefly luciferase gene (pGL3-firefly; Promega), with Lipofectamine 2000 (Invitrogen) transfection reagent, following the manufacturer’s instructions. Each construct was tested in triplicate in each of three separate transfections. Seventy-two hours after transfection, the activities of firefly and NanoLuc luciferase were measured using the Nano-Glo Dual-Luciferase reporter assay system (Promega, #N1610). The NanoLuc luciferase activity was normalized with firefly luciferase activity for each transfection.

### Microscopy

Stained samples were imaged using a Nikon Eclipse TiE inverted microscope with a Photometrics charge-coupled device camera. The images were deconvolved and uniformly processed using NIS-Elements software.

## Additional information

**How to cite this article:** Sarkar, M. K. *et al.* An Xist-activating antisense RNA required for X-chromosome inactivation. *Nat. Commun.* 6:8564 doi: 10.1038/ncomms9564 (2015).

## Supplementary Material

Supplementary Information Supplementary Figure 1 and Supplementary Table 1

## Figures and Tables

**Table 1:** List of sequences tested for promoter and enhancer activity.

**Promoter/enhancer**	**Sequence**
*Xist* P1 promoter	Xist bp −672 to +52
*RepA* promoter	Xist bp 79 to 320
*XistAR* promoter 1	Xist bp 3,000 to 2,750
*XistAR* promoter 2	Xist bp 3,500 to 2,800
*XistAR* enhancer 1	Xist bp 2,000 to 1,380
*XistAR* enhancer 2	Xist bp 2,520 to 1,981
*XistAR* enhancer 3	Xist bp 3,000 to 2,461
*XistAR* enhancer 4	Xist bp 3,500 to 2,800
*XistAR* enhancer 5	Xist bp 3,000 to 1,380
