# Supplementary material for: An Xist-activating antisense RNA required for X-chromosome inactivation
Source: Nat Commun. 2015 Oct 19;6:8564. doi: 10.1038/ncomms9564 (PMC4616153; doi:10.1038/ncomms9564)
Supplement: Supplementary Information — Supplementary Figure 1 and Supplementary Table 1 [file ncomms9564-s1.pdf]

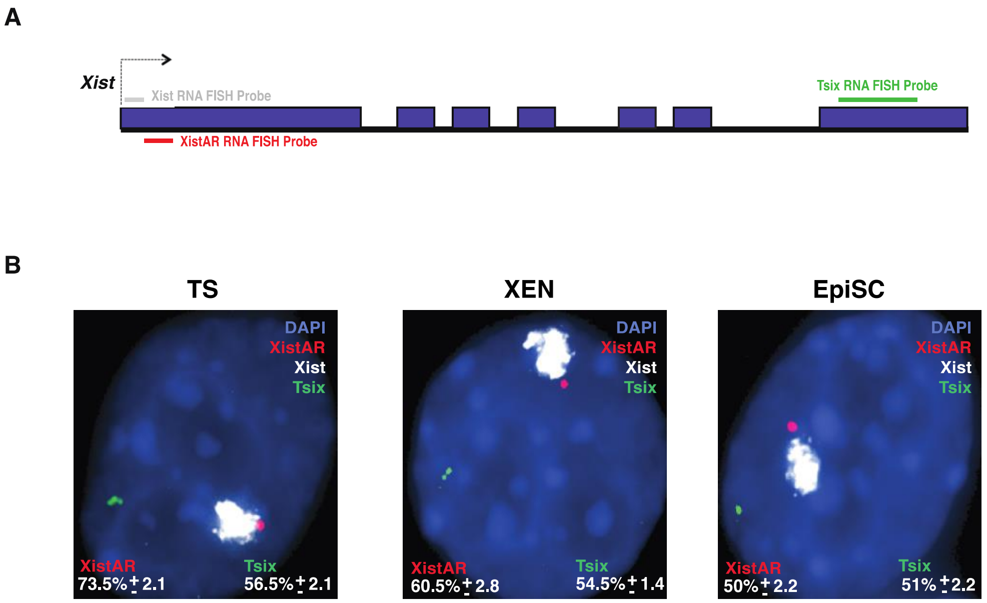

Supplementary Fig. S1. Detection of XistAR, Tsix, and Xist RNAs in TS cells, XEN cells, and EpiSCs by RNA FISH. (A) Schematic representation of the Xist locus and location of the different strand specific RNA FISH probes used. **(B)** Strand-specific RNA FISH detection of Xist (white), XistAR (red) and Tsix (green) RNAs in TS, XEN and EpiSC cell lines. Nuclei are stained blue with DAPI. Two different TS, XEN and EpiSC cell lines were stained and >100 nuclei counted in each cell line. Numerical values in the images indicate the percentage of nuclei that display XistAR and Tsix RNA signals. ± standard deviation.

Supplementary Table 1: List of Primers

Primer	Sequence	Use	Figure(s)
AUAP	GGCCACGCGTCGACTAGTAC	PCR	1, 3
XistF7	(AUAP)-GCTCCAGCCATGTTTGCTCGTT	RT	3
XF13	(AUAP)-GCCATGTTTGCTCGTTTCCC	RT	3
XF718	(AUAP)-CGGGGCGCTTGGTGGATGGAAAT	RT	1, 3
XF1440	(AUAP)-GTGGCACATGCTCTTGAATG	RT	3
XR1648	GGCAACCAACACTTCCACTT	PCR	3
XR2530	AGCTCGGTGGATGAGTTTGA	Nested PCR	3
XR2640	AAAAGCAAAGCCTGAACTGG	PCR	3
XR1502	AACCACTAGAGGGCAGGTCA	Nested PCR	1, 3
XR9572	CCAGGCAATCCTTCTTCTTG	PCR	6
XF9229	GACAACAATGGGAGCTGGTT	RT, PCR	6
XR926	CGTATCACGCAGAAGCCATA	PCR	3, 6
XR883	CCCTGAACACCCACTCAGTT	Nested PCR	3, 6
XF1851	GGAGAGCGCATGCTTGCAAT	PCR	3
XF2121	GCCTCTGATTTAGCCAGCAC	Nested PCR	3
XF2296	TATTTTGGATGCCAACGACA	Nested PCR	3
XF953	(T7)-AATTAGGACACCGAGGAGCA	FISH	1, 6
XR1440	(T3)-CACAAAGCCCTTTGCGTTAT	FISH	1, 6
XistF	CAAGAAGAAGGATTGCCTGGATTT	PCR	6
XistR-biotin	GCGAGGACTTGAAGAGAAGTTCTG	RT, PCR	6
Xist-seq	CAAACAATCCCTATGTGA	PCR	6
Utx-F-biotin	CCAAAAGCATTATCTGCATACCA	PCR	6
Utx-R	CCAAACCAAGACCATATAAAAAGG	RT, PCR	6
Utx-seq	TAGAACTTCCTTCAGGC	Pyroseq.	6
Rnf12-F-biotin	TGCAGCCAACAAGTGAAATTCC	PCR	6
Rnf12-R	TATCTGCTGTCTCAGGGTCACATG	RT, PCR	6
Rnf12-seq	TAGAACTTCCTTCAGGC	Pyroseq.	6
Atrx-F	ATAGCTTCAGATTCTGATGAAACC	PCR	6
Atrx-R-biotin	ACATCGTTGTCACTGCCACTT	RT, PCR	6

Atrx-seq	TAAGCTCAGATGAAAAGA	Pyroseq.	6
Pdha1-F-biotin	AGCAATCTTGCAAGTGTTGAAGAA	PCR	6
Pdha1-R	TTTTCAAGCCTTTTGTTGTCTGG	RT, PCR	6
Pdha1-seq	TAGAACTTCCTTCAGGC	Pyroseq.	6
P1	(AUAP)-GGCCAGGCTGGAGCTCTCAG	RT	6
P2	ACAAAGATTGGGCTGTCGAG	PCR	6
P3	GCACAACCCCGCAAATGCTA	Nested PCR	6
D1	(AUAP)-CGGGGCGCTTGGTGGATGGAAAT	RT	6
D2	CCCTGCTGTGCTCAGATCAA	PCR	6
D3	CCCTGAACACCCACTCAGTT	Nested PCR	6
XF720	GGGCGCTTGGTGGATGGAAAT	RT-qPCR	6
XR883	CCCTGAACACCCACTCAGTT	RT-qPCR	6
DR1	CCCTGCTGTGCTCAGATCAA	RT-qPCR	6
PF3	TGGGAAAGCAAAATCTCAGG	RT-qPCR	6
XR941	TGGCTCGAGAATAGCCGTAT	RT-qPCR	6
XF1440	GTGGCACATGCTCTTGAATG	RT-qPCR	6
XR1648	GGCAACCAACACTTCCACTT	RT-qPCR	6
XF2350	AAAACCGAAGTGATTGTTTTCAAA	RT-qPCR	5,6
XR2483	TTTGAGTATCATCTGCCAAAAA	RT-qPCR	5,6
TF5	GTTCATGCGTGCGCACGTGTA	RT, PCR	6
TR732	GGAGAGCGCATGCTTGCAAT	RT,PCR	5,6
TF350	CCTGCAAGCGCTACACACTT	PCR	5
